# The Neural Responses of Visual Complexity in the Oddball Paradigm: An ERP Study

**DOI:** 10.3390/brainsci12040447

**Published:** 2022-03-27

**Authors:** Rui Hu, Liqun Zhang, Pu Meng, Xin Meng, Minghan Weng

**Affiliations:** School of Design, Shanghai Jiao Tong University, 800 Dong Chuan Road, Shanghai 200240, China; hurui_onssa@sjtu.edu.cn (R.H.); mengpu0312@sjtu.edu.cn (P.M.); mx714540581@sjtu.edu.cn (X.M.); wengmh44@sjtu.edu.cn (M.W.)

**Keywords:** visual complexity, event-related potentials, oddball paradigm, visual mismatch negativity

## Abstract

This research measured human neural responses to images of different visual complexity levels using the oddball paradigm to explore the neurocognitive responses of complexity perception in visual processing. In the task, 24 participants (12 females) were required to react to images with high complexity for all stimuli. We hypothesized that high-complexity stimuli would induce early visual and attentional processing effects and may elicit the visual mismatch negativity responses and the emergence of error-related negativity. Our results showed that the amplitude of P1 and N1 were unaffected by complexity in the early visual processing. Under the target stimuli, both N2 and P3b components were reported, suggesting that the N2 component was sensitive to the complexity deviation, and the attentional processing related to complexity may be derived from the occipital zone according to the feature of the P3b component. In addition, compared with the low-complexity stimulus, the high-complexity stimulus aroused a larger amplitude of the visual mismatch negativity. The detected error negativity (Ne) component reflected the error detection of the participants’ mismatch between visual complexity and psychological expectations.

## 1. Introduction

In visual processing, like any information-processing system, the visual cortex is limited in the quantity of information it can process at each moment in time [1]. From the psychological perspective, it regards human’s subjective perception of complexity as the research object, and the main research direction is visual complexity. Visual complexity is an important concept, but it is difficult to define. “Complexity” is used in two different ways. On one hand, there is the view that the complexity is the *Quality* that makes the system complex. On the other hand, it has also been thought that some things are more complex than others. In this case, complexity is used as *Quantity* [2].

To understand visual complexity, Koffka thought that the brain acted on a visual input to modify the resulting perception of an ideal or optimum. Even in the absence of sensory input, brain dynamics make the trace change over time [3]. Berlyne described visual complexity as being affected by a combination of factors [4] and argued that the arousal potential of a stimulus (thought to be related to complexity and novelty) was related to its hedonic value through an inverted U-shaped function [5]. Some scholars also found that participants’ descriptions of image complexity were consistent with a multi-dimensional representation of visual complexity [6], an implicit measure of cognitive load that may correlate to visual complexity [7]. Thus far, most studies have considered visual complexity as a one-dimensional attribute, and some studies have proposed two dimensions of visual complexity, namely the number and variety of elements and the organization and grouping of elements, to explain differences beyond one dimension [8].

In the cognitive research of visual complexity, Silva believed that complexity had a dominant relationship with cognitive load. An attentional-based definition of complexity perception was proposed [9]. Subsequently, researchers focused on the attention processing and added the task complexity dimension into their research to explore the relationship between human visual cognition and cognitive load under different complexity conditions [10,11].

In studies of neural representations of visual complexity, research focused on analyzing the findings of mapping brain-activation features, namely, the findings of representations of extended brain regions whose mean activity varied under experimental conditions [12]. Neural characteristics of visual complexity were caused by stimuli with different complexity features. Therefore, research has usually involved the measuring event-related potentials (ERPs) [13,14,15,16]. The visual processing of complex shapes is more error-prone than that of simple shapes, manifesting as the set size effects of slow waves and posterior P2 [17]. The more complex stimuli caused a longer viewing duration, and greater amplitudes of the anterior N2 component and the late positive LPP component [16]. In a study of scene images [18], under high-complexity conditions, the electroencephalogram (EEG) signal drift rate of the early visual cortex was most significantly affected by the late ERP amplitude, which suggested that when processing high-complexity natural scene images, the feedforward activities had insufficient information and needed more feedback interaction drive. Researchers also investigated neuronal activity specifically associated with retention in visual short-term memory [19]. The sustained posterior contralateral negativity during the retention interval was larger for complex objects than for simple objects.

The previous studies have examined particular features of image complexity, or real images such as scene images. Our study considered complexity as an independent feature and determined which neural responses are involved in the modulation of complexity on artistic images in the human visual system. The following four hypotheses are discussed based on the visual ERP component features, along with corresponding neural mechanisms and cognitive implications.

**Hypothesis** **1** **(H1).** *In the early stage of the visual processing, complexity will lead to attention allocation, manifesting as the appearance of the N1 component, but may not create substantial variations in P1 amplitudes*.

**Hypothesis** **2** **(H2).** *When the participants respond to the stimuli, the presence of response inhibition in the processing of complexity may be reflected in the form of the N2 component.*

**Hypothesis** **3** **(H3).** *In the objective observation, differences in complexity may cause processing in visual mismatch negativity (vMMN).*

**Hypothesis** **4** **(H4).** *Among the feedback-related ERPs, an automatic error identification-related ERP, which is error negativity (Ne or ERN), or a subsequent controlled error-identification and task-reassessment process, which is error positivity (Pe), may be found.*

In the following sections, we briefly review the neural correlates of visual processing in relation to complexity to support our hypotheses about the neural representations of visual complexity in this study.

The visual P1 and N1 components of visual evoked potentials (VEPs) have generally been connected with the early stages of visual processing. Consecutive time windows, early categorization (P1, around about 100 ms), and stimulus recognition (N1, around about 150 ms) [20] can be used to describe the chronological course of visual information processing.

P1 and N1 are early ERP components that predominantly reflect external processes governed by physical stimulus qualities, not cognitive processes [21]. The brightness of the visual stimulus, for example, influences both visual P1 and N1, and the stimulus invokes the task that the participant is completing [22] irrespective of the stimulus.

P1 is produced in extrastriate areas [23] and has a latency of approximately 100 ms [24]. P1 has been widely investigated via emotional images [25] and was previously assumed to be associated with very rapid neural activity processing faces [26,27,28,29]. P1 face sensitivity is essentially a response to low-level visual cues of the stimuli, according to further studies [30]. P1 exhibits early attentional modulation [31], and promotes early spatial-selectivity processing of stimuli presented at attended targets [32,33,34]. The regulation of non-spatial attention by P1 has also been verified, and P1 amplitude may be altered by color-based attention when attended and unattended colors are competing [35]. 

The N1 component is related to characterizing the sequence of neural events from early attentional mechanisms that foster perceptual feature extraction both in anterior and posterior areas [36]. For the selective attention effects, these can be dissociated by the anterior scalp distributed spatial-based attention effect and posterior scalp distributed object-based attention effect [37,38]. Further studies confirmed the operation of a voluntary discrimination process of N1, which demonstrated its sensitivity to physical stimulus factors, and can be elicited by color- or form-based discriminations, consistent with the hypothesis that the visual N1 component reflects the operation of a discrimination process within the focus of attention [39,40].

In studies on complexity perception, the influence of complex stimuli on P1 is still unclear, although studies have indicated that in both target and non-target situations, the occipital N1 amplitude stimulus was larger for complex stimuli than simple stimuli [41]. Analogously, we hypothesized that although variations in artistic picture complexity may not generate significant differences in P1, they should induce significant differences in N1 amplitudes.

The N2 component is a negative wave peaking between 200 and 350 ms after stimulus onset. The N2 component reflects cognitive control, novelty, and sequential matching mechanisms [42,43]. Regarding the visually evoked N2 component, it is now divided into two main subcomponents in studies, namely N2b [44,45] and N2c [46,47]. The anterior N2, which is N2b, has a central scalp distribution and is accompanied by P3a, considered to be indices for different stages of mismatch detection. N2c is also the posterior N2; its latency is correlated with reaction time, located posteriorly in the visual mode. The N2c component was thought to reflect a subprocess of stimulus classification. In the two-stimulus oddball paradigm, rare visual targets elicit a larger N2 over the parietal, temporal, and occipital scalp, followed by a larger P3b [48], whereas for the N2 novelty effect, the complex novel stimuli elicited a larger frontal N2 [49], revealing that the frontocentral N2 was sensitive to visual novelty and attended mismatch visual template. Thus, there are two functional sources of the N2 component: one is elicited by visual stimuli and has the maxima over frontal or central scalp sites, and the other is control-related with the possible exception of the feedback-related negativity, which is independent of mismatch detection [43]. In this study, we hypothesized that a control-related frontocentral N2 component may be evoked when the “conflict” occurs between the reaction responses and the expectation of stimulus.

Studies have shown that the human brain can even detect small visual changes, especially if such changes violate automatic expectations [50], and have defined the deviant minus standard difference potential as a mismatch negativity component (MMN). The MMN response is widely considered as a perceptual prediction error signal both in auditory modality and visual modality [51,52]. Previous studies have carried out tasks with the visual materials of orientation [53,54,55], color [56,57,58], pattern [59,60], and facial categories [9,10,11], and proved the correlation between visual MMN (vMMN) and the above individual characteristics. It has also been investigated for feature conjunctions, object-related deviances, and the violation of sequential regulations [53,54]. vMMN has been confirmed in the cognitive process of automatic stimulus discrimination [61,62], and research has shown the automatic categorization processes are based on fairly complex stimulus representation [63,64]. In this study, we planned to use the oddball paradigm in which non-repetitive stimuli appear randomly, and assumed that the perception processing of visual complexity will provoke the automatic discrimination effect.

In the ERPs of incorrect choice reactions, the error negativity, or error-related negativity (Ne or ERN), is a negative potential with a frontocentral maximum and subsequent positive potential, whereas the centro-parietal maximum is error positivity (Pe) [65]. Researchers considered the Ne component of error detection [66], error inhibition [67], and monitoring processes that are sensitive to response conflict [68], or the production of a reward-prediction error signal for the adaptive modification of behavior [69]. In the experimental design of this study, we asked participants to respond during the task, expecting to find features and explanations related to complexity cognition and discrimination processes in the ERPs generated by the error deviations of trials.

The measurement of human visual complexity contributes to expanding the research dimensions of exploring the neural responses of the human visual system when processing visual objects of different complexities. Previous studies have contributed ERP responses on stimuli of different image properties relating complexity. This study aimed to obtain neural responses to visual complexity by presenting an oddball paradigm task of artistic images with different complexity levels to better understand the neurocognitive modulations of complexity perception in visual processing.

## 2. Materials and Methods

### 2.1. Participants

The experiment recruited 24 college students (12 females, M = 23.67 years, SD = 1.01) from Shanghai Jiao Tong University. All participants were right-handed and had normal or corrected to normal vision. No participants reported a history of psychiatric or neurological diseases. All participants read the experimental procedures and signed the informed consent, and also authorized the usage of the data generated by their participation. They received financial compensation for their participation. The study followed the rules of the Declaration of Helsinki of 1975, revised in 2013, and was reviewed and approved by the Institutional Review Board for Human Research Protections (IRB. HRP) of Shanghai Jiao Tong University.

### 2.2. Materials

**Figure 1:**
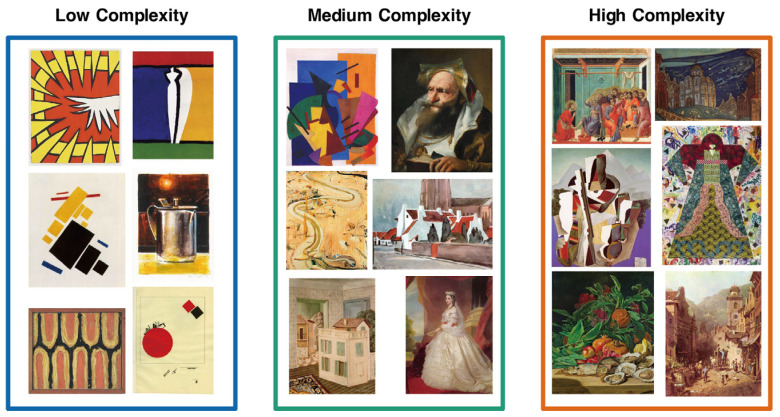
Examples of stimuli under low-, medium-, and high-complexity conditions. A total of 254 images were used for the experiment. For the low-complexity condition group, the complexity score range was 1–33, the medium-complexity condition group score range was 34–66, and the high-complexity condition group score range was 67–100.

To achieve reliable results, we chose the open-source SAVOIAS image dataset provided by Elham Saraee et al. [70] as the source of stimuli, which is the latest image dataset on complexity. It was evaluated using a forced-choice pairwise crowdsourcing process and validated using unsupervised methods, which have quantitative and credibility advantages. The dataset contained 1420 images in 7 categories; each image had an absolute score of (0,100). These stimuli in our study were selected from the art category in the SAVOIAS dataset, with a total of 254 images after scoring by experts. In the expert review, we employed 3 specialists in the domains of art, visual cognition, and computer vision to evaluate all of the images, rejecting images with text, images blended with real sceneries and art forms, and images with high emotional arousal. Then, we categorized the selected images into three complexity levels according to their scores to match the oddball diagram criteria. Examples from each stimulus complexity condition are shown in Figure 1.

### 2.3. Procedure

**Figure 2:**
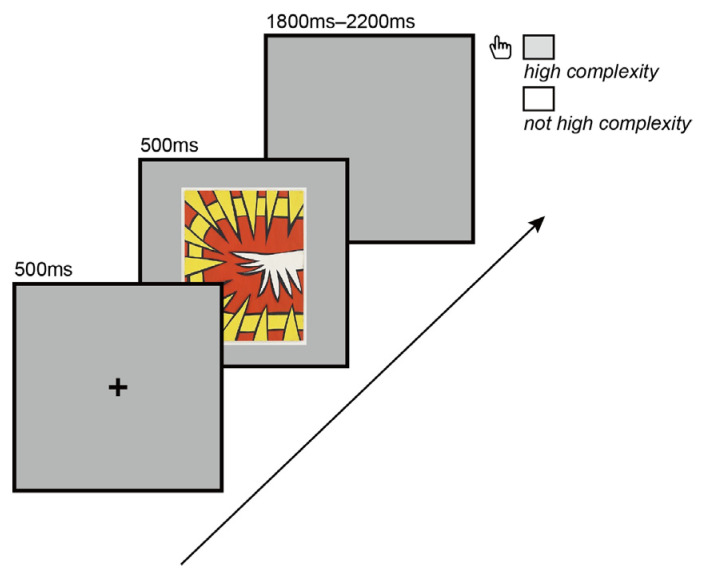
The experimental design of the task is shown in Figure 2. Each image in the task was presented for 500 ms. There was a random blank interval of between 1800 and 2200 ms between every two images to allow the participants to judge the image’s complexity. Participants were told to press the space bar when the image met the high-complexity condition in their expectations. If not, no response was required.

The experiment program was written and displayed in the E-Studio 3.0 software (Psychology Software Tools, Inc., Sharpsburg, MD, USA). The program contained 7 blocks, including a pre-experimental block; 35 images were shown in each block, with a total of 245 images. In addition, the participants were shown 9 sample images, 3 for each condition, before the pre-experiment. Participants received feedback on the pre-experimental trials. We used a three-stimulus visual oddball paradigm to modulate the ERP components we mentioned in the hypotheses. The proportion of the standard stimulation (low-complexity stimuli), non-target stimulation (medium-complexity stimuli), and target stimulation (high-complexity stimuli) was 5:1:1. Each image in the task was presented for 500 ms. There was a random blank interval of between 1800 and 2200 ms between every two images to allow the participants to react to the complexity of the image. Participants were instructed to press the space bar when the image met the high-complexity condition in their expectations. If not, no response was required. The experimental design is shown in Figure 2. The experiment was carried out in a quiet laboratory with suitable indoor light.

### 2.4. Data Recording and Analysis

The EEG was recorded from 64 Ag/AgCl electrode scalp sites according to the 10–20 system for electrode placement using the ANT Neuro eego™ mylab (ANT Neuro, Hengelo, The Netherlands) wave-guard EEG cap, and a 64-channel eego amplifier (16 kHz) was matched. The ground electrode was placed on the scalp at a site equidistant between Fpz and Fz, and the reference electrode at CPz. The sampling rate was 500 Hz and all electrode impedances were kept below 5 kΩ [71]. Participants put on the electrode cap and kept 60 cm from the displayer after being told and reported comprehension of the task book. The participants could start the task after the researchers observed that the EEG signal recording was effective within the ANT Neuro eego^TM^ mylab software.

The EEG analysis was conducted with MATLAB_R2021a using the EEGLAB v2021.1 toolbox [72]. In EEGLAB, we used the MNI coordinate file for the BEM dipfit model to import channel locations. After importing the channel locations, we deleted the EOG channel and interpolated the bad electrodes. Due to the variation in the quality of the data supplied by each individual, the most interpolated had three faulty electrodes, whereas the least had none. M1 and M2 were used as the reference channels to re-reference the data. We filtered the data with a 0.5 to 30 Hz bandpass. Then, we used the open-source toolbox ERPLAB v8.10 [73] for ERP extraction and analysis. We created an event list and extracted bin-based epochs with a time window of −200 to 800 ms. Before the data onset, a baseline with a latency time of 200 ms was used for correction. Independent component analysis (ICA) was used to identify and remove stereotypical artifacts using the Runica algorithm from the EEGLAB toolbox. Two components rejected were vertical and horizontal eye movement components; the components were marked by inspection and rejected automatically by ICA. To eliminate electromyogram and other artifacts from the ERP data, we used a rejection threshold of an extreme value of −100 to 100 μV to reject marked epochs. During the preprocessing, we eliminated 1 piece of invalid data that was not completely marked in recording and 3 abnormal data with excessive overall signal drift or artifacts. After processing the data, 508 epochs of each data were used in the study.

**Figure 3:**
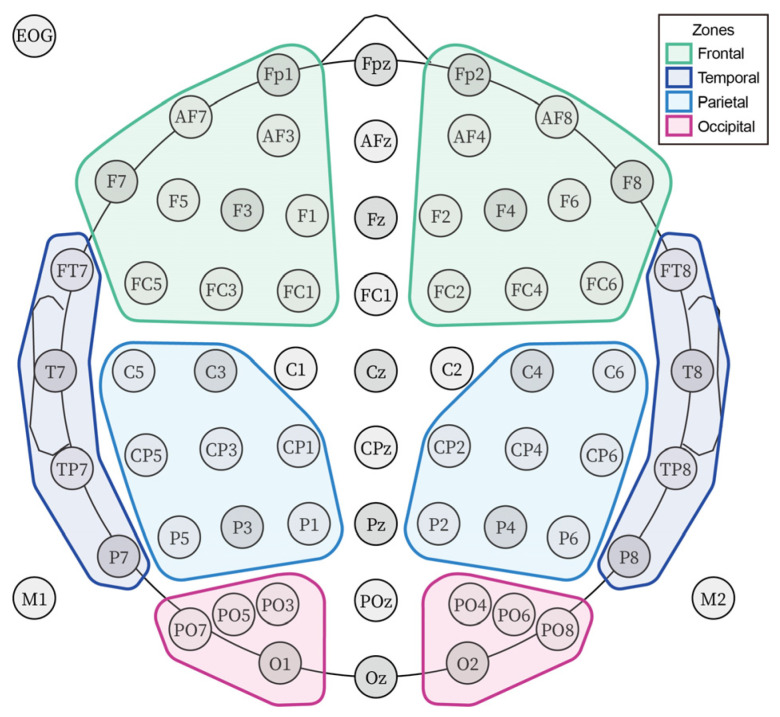
Grouping map of the scalp within the electrodes. The scalp zones were divided as follows. The left frontal zone: FP1, AF3, AF7, F1, F3, F5, F7, FC1, FC3, FC5. The right frontal zone: FP2, AF4, AF8, F2, F4, F6, F8, FC2, FC4, FC6. The left temporal zone: FT7, T7, TP7, P7. The right temporal zone: FT8, T8, TP8, P8. The left parietal zone: C3, C5, CP1, CP3, CP5, P1, P3, P5. The right parietal zone: C4, C6, CP2, CP4, CP6, P2, P4, P6. The left occipital zone: PO3, PO5, PO7, O1. The right occipital zone: PO4, PO6, PO8, O2.

For the ERP averaging, we used ERPLAB to compute average ERPs and generated the grand average ERP dataset. To examine the interactive effect between the ERP levels and brain zones, we divided the channels into 4 zones (frontal, temporal, parietal, occipital). The voltages of all regions were averaged for analysis. Figure 3 shows the electrode division of the zones. We also looked at the hemispheric impacts in the interaction effect to further understand and discuss the ERP components. Based on the inspection of the grand-average ERP waveform, we selected the time windows P1 (80–120 ms), N1 (140–200 ms), N2 (170–300 ms), and P3 (250–400 ms). To generate vMMN in our research, we applied the target-minus-standard method [41]. We selected the 150–400 ms time window for the vMMN [74]. The epochs of Ne and Pe were extracted by the response marks with a time window from −200 ms to 800 ms; a pre-response baseline of 200 ms was used [75]. We selected the 0–100 ms time window for the Ne component and 100–250 ms for the Pe component [65].

All analyses were conducted using SPSS v26.0.0.0. We performed repeated-measures ANOVA analysis on the data. The Greenhouse–Geisser method was used to calculate the *p*-values for the deviations once the spherical assumption was rejected. Bonferroni adjustments were used to perform post hoc t-tests for multiple comparisons.

## 3. Results

### 3.1. Behavioral Analysis

A normality test was completed on the matching between the participants’ judgment on the visual complexity and the complexity level of the image. The result of the Shapiro–Wilk test (Correct: M = 194.65, SD = 21.53, *p* = 0.022; Error: M = 60.65, SD = 21.65, *p* = 0.04) indicated that the data were not normal, so a nonparametric test was selected. The result of the Wilcoxon matched-pairs signed-rank test showed that participants’ correct responses were significantly higher than wrong responses (Z = −3.92, *p* < 0.001). For correct-response trials, the mean reaction time was 2375.33 ms, and the standard deviation was 71.81 ms. The mean reaction time of error reaction trials was 1858.43 ms, with a standard deviation of 301.4 ms.

### 3.2. Event-Related Potentials

#### 3.2.1. P1

**Figure 4:**
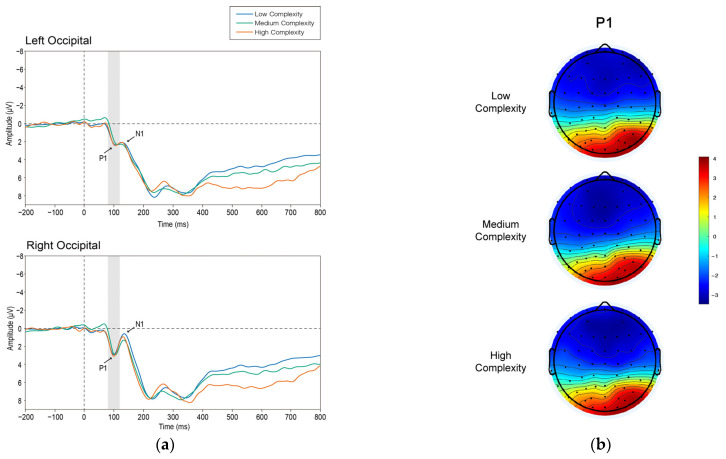
(**a**) The grand average waveform of the P1 component. The wave in blue represents the low-complexity condition, the wave in green represents the medium-complexity condition, and the wave in red represents the high-complexity condition. The gray bars indicate the time window of the P1. (**b**) The mean voltage 2D topography map of the P1 component. The color red means higher positive amplitudes, while the color blue means more negative amplitudes.

Figure 4 displays the grand average waveform (a) and the mean voltage scalp map (b) of P1.

We examined the P1 component in the occipital region. A repeated-measures ANOVA with hemisphere (left and right) and complexity (low, medium, high) was used. The main effects of hemisphere were significant in the ANOVA (F_1, 19_ = 5.851, *p* = 0.026, η^2^ = 0.235). The interaction between hemisphere and complexity was not statistically significant (F_2, 38_ = 0.94, *p* = 0.4, η^2^ = 0.047). The mean peak amplitude in the occipital region of the right hemisphere was significantly greater than that in the occipital region of the left hemisphere (MD = 1.047, SE = 0.433, *p* = 0.026), the mean amplitude of the right hemisphere was 3.222 μV (SD = 0.542), and the mean amplitude of the left hemisphere was 4.269 μV (SD = 0.579).

Regarding the occipital N1, a repeated-measures ANOVA was introduced, and hemisphere (left and right) and complexity (low, medium, high) were used. The main effects of hemisphere were significant in the ANOVA (F_1, 19_ = 4.654, *p* = 0.044, η^2^ = 0.197). The interaction between hemisphere and complexity was not significant (F_2, 38_ = 1.395, *p* = 0.26, η^2^ = 0.068). The mean peak amplitude in the occipital region of the right hemisphere was significantly lower than that in the occipital region of the left hemisphere (MD = −1.448, SE = 0.671, *p* = 0.021). It is worth noting that the mean amplitude of N1 remained positive, with 0.871 μV (SD = 0.506) in the right hemisphere and 2.319 μV (SD = 0.795) in the left hemisphere.

#### 3.2.2. N2

Regarding the N2 component, a repeated-measures ANOVA was introduced with hemisphere (left and right), zone (frontal and temporal), and complexity (low, medium, high). The analysis indicated a significant main effect of the zone (F_1, 19_ = 42.442, *p* < 0.001, η^2^ = 0.691) and complexity (F_2, 29_ = 6.975, *p* = 0.006, η^2^ = 0.269). The interaction effect was significant between zone and complexity (F_2, 38_ = 9.659, *p* < 0.001, η^2^ = 0.337).

**Figure 5:**
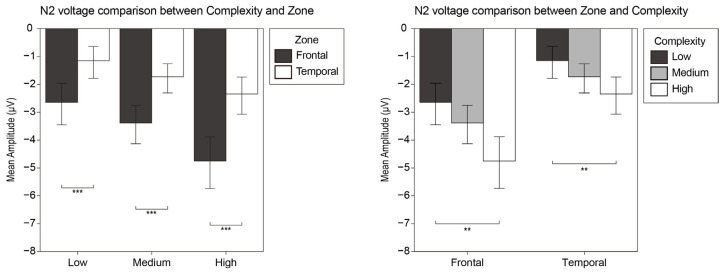
Bar plots of the interaction effect of the N2 component. Values plotted are means ± standard errors. Stars indicate significance levels: ** = *p* < 0.01 and *** = *p* < 0.001.

A post hoc analysis was undertaken with the interaction effect between zone and complexity (Figure 5). The result showed that the N2 voltage in the frontal zone was significantly higher than in the temporal zone under all the low-, medium-, and high-complexity conditions (MD = 1.493, SE = 0.279, *p* < 0.001; MD = 1.658, SE = 0.264, *p* < 0.001 and MD = 2.407, SE = 0.38, *p* < 0.001). Additionally, the N2 voltage under the high-complexity condition was significantly higher than that under the low-complexity condition in the both the frontal zone and temporal zone (MD = 2.104, SE = 0.524, *p* = 0.002 and MD = 1.19, SE = 0.362, *p* = 0.012). 

#### 3.2.3. P3

**Figure 6:**
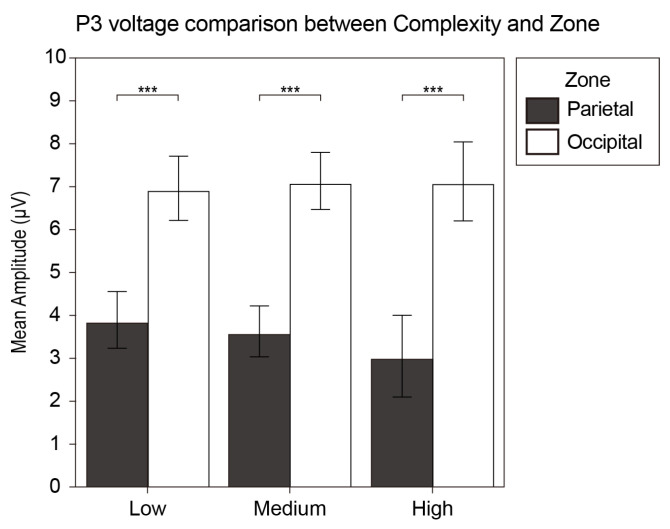
Bar plots of the interaction effect of the P3 component. Values plotted are means ± standard errors. Stars indicate significance levels: *** = *p* < 0.001.

Regarding the P3 component, we also conducted a repeated-measures ANOVA analysis with hemisphere (left and right), zone (parietal and occipital), and complexity (low, medium, high). We found that the zone factor had a significant main effect (F_1, 19_ = 58.927, *p* < 0.001, η^2^ = 0.756). For the interaction effect between zone and complexity (F_1, 24_ = 18.777, *p* < 0.01, η^2^ = 0.497), the follow-up post hoc analysis revealed P3 voltage in the occipital zone was significantly greater than that in the parietal zone under all the low-, medium-, and high-complexity conditions (MD = 3.062, SE = 0.427, *p* < 0.001; MD = 3.497, SE = 0.46, *p* < 0.001 and MD = 4.058, SE = 0.52, *p* < 0.001). Figure 6 shows the statistics of the P3 component.

#### 3.2.4. vMMN

To explore whether the visual complexity is a visual feature that causes the visual mismatch negativity (vMMN), we calculated the difference wave of the participants under all complexity conditions with a latency of 150–400 ms.

We undertook a repeated-measures ANOVA analysis with hemisphere (left and right), zone (frontal, temporal, parietal, occipital), and difference in complexity (high/low, high/medium, medium/low). The analysis indicated that the zone and the difference in complexity had the main effect (F_3, 17_ = 6.921, *p* = 0.003, η^2^ = 0.55 and F_2, 18_ = 6.919, *p* = 0.006, η^2^ = 0.435). Additionally, the zone factor and the difference in complexity factor had a significant interaction effect (F_6, 14_ = 6.165, *p* = 0.002, η^2^ = 0.725).

**Figure 7:**
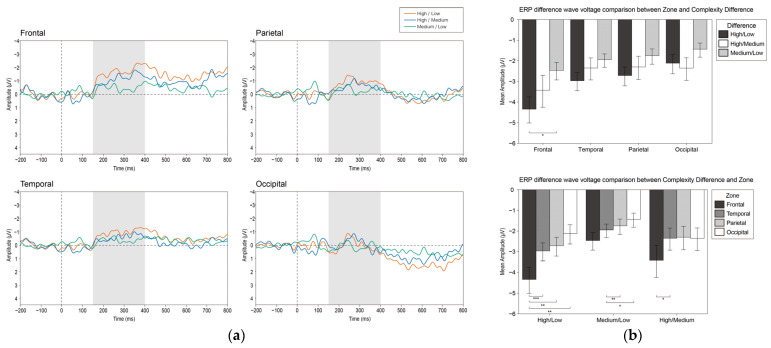
(**a**) The grand average waveform of the vMMN. The wave in blue represents the high/medium condition, the wave in green represents the medium/low condition, and the wave in red represents the high/low condition. The gray bars indicate the time window of the vMMN. (**b**) The bar plots of the interaction effect of the vMMN. Values plotted are means ± standard errors. Stars indicate significance levels: * = *p* < 0.05, ** = *p* < 0.01, *** = *p* < 0.001.

A post hoc analysis was performed, and the result revealed that the high/low difference wave in the frontal zone was significantly greater than that in the temporal, parietal, and occipital zone (MD = 1.377, SE = 0.295, *p* = 0.001; MD = 1.634, SE = 0.416, *p* = 0.005 and MD = 2.231, SE = 0.508, *p* = 0.002). The medium/low difference wave in the temporal zone was significantly greater than that in the parietal and occipital zone (MD = 0.711, SE = 0.232, *p* = 0.038 and MD = 1.03, SE = 0.264, *p* = 0.006). Moreover, the high/medium difference wave in the frontal zone was significantly greater than that in the temporal zone (MD = 1.086, SE = 0.344, *p* = 0.031). In the frontal zone, the high/low difference wave was significantly greater than the medium/low difference wave (MD = 1.889, SE = 0.706 *p* = 0.045). Figure 7 displays the grand average waveform (a) and the analysis results (b) of the vMMN.

#### 3.2.5. Ne and Pe

**Figure 8:**
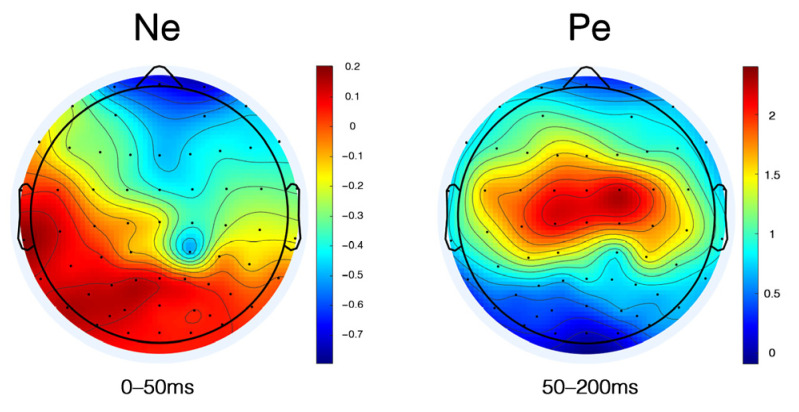
The grand scalp 2D topography map of Ne and Pe. The time window of the mean amplitudes of Ne was 0–50 ms, and the time window of the mean amplitudes of Pe was 50−200 ms. The color red means higher positive amplitudes, whereas the color blue means more negative amplitudes.

In the task, participants were required to judge “whether the image meets the high complexity” after viewing each image. To further analyze the difference between the complexity grade of the image and the participants’ psychological complexity judgment, we calculated the error negativity (Ne) and error positivity (Pe) at the FPz, Fz, FCz, and Cz electrodes since the anterior cingulate cortex (ACC) has been shown to respond to conflict and error detection [68]. There were 1206 trails of Ne and 3887 trails of Pe. Misses and false alarms were contained in the Ne trails. Figure 8 displays the mean voltage scalp map of Ne and Pe.

We conducted repeated-measures ANOVA analysis with the wave type (correct wave, error wave, difference wave) and electrode (Fpz, Fz, FCz, Cz) for Ne and Pe. 

**Figure 9:**
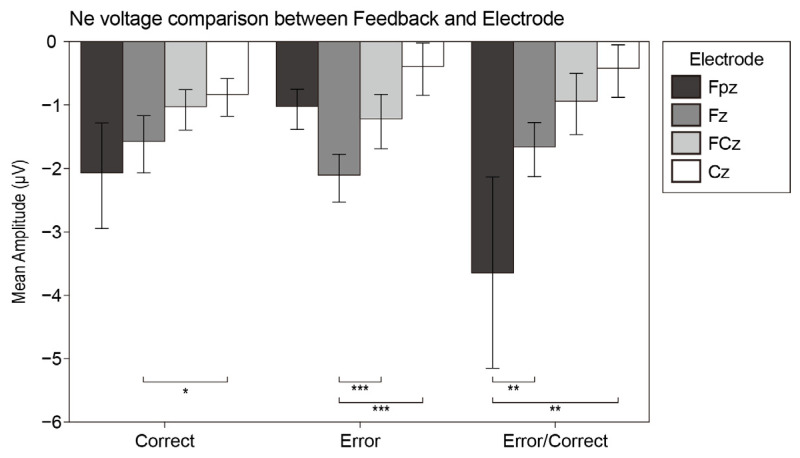
The bar plots of the interaction effect of Ne. Values plotted are means ± standard errors. Stars indicate significance levels: * = *p* < 0.05, ** = *p* < 0.01, *** = *p* < 0.001.

For the Ne component, the electrode factor had a significant main effect (F_3, 16_ = 9.095, *p* = 0.001, η^2^ = 0.63). Furthermore, the interaction effect was significant (F_6, 13_ = 9.192, *p* < 0.001, η^2^ = 0.809). The post hoc analysis reported that the amplitude of Fz was significantly higher than that of Cz at the correct wave (MD = 0.73, SE = 0.234, *p* = 0.035). The amplitude of Fz was significantly higher than that of FCz and Cz at the error wave (MD = 1.691, SE = 0.367, *p* = 0.001 and MD = 0.815, SE = 0.148, *p* < 0.001). The amplitude of Fpz was significantly higher than that of Fz and Cz at the Ne wave (MD = 1.218, SE = 0.269, *p* = 0.002 and MD = 0.709, SE = 0.185, *p* = 0.007). Figure 9 displays the analysis results of the Ne component.

**Figure 10:**
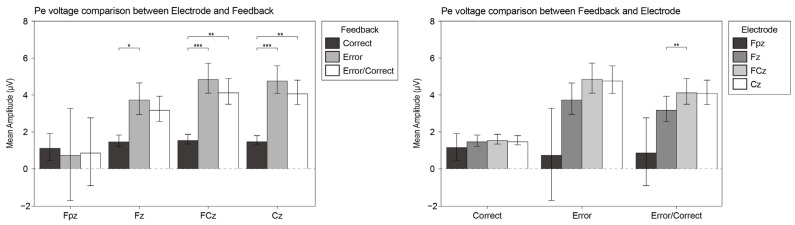
The bar plots of the interaction effect of Pe. Values plotted are means ± standard errors. Stars indicate significance levels: * = *p* < 0.05, ** = *p* < 0.01, *** = *p* < 0.001.

For the Pe component, the wave type factor had a significant main effect (F_1, 19_ = 5.425, *p* = 0.031, η^2^ = 0.222) and the interaction effect was significant (F_1, 19_ = 5.131, *p* = 0.035, η^2^ = 0.213). The post hoc analysis demonstrated the amplitude of FCz was significantly higher than that of Fz at the Pe wave (MD = 0.942, SE = 0.254, *p* = 0.009). On the Fz, FCz and Cz electrodes, the amplitude of the error wave was significantly higher than the correct wave (MD = 2.226, SE = 0.723, *p* = 0.016; MD = 3.288, SE = 0.663, *p* < 0.001, and MD = 3.302, SE = 0.726, *p* = 0.001, respectively). Furthermore, on the FCz and Cz electrodes, the amplitude of the Pe wave was significantly higher than the correct wave (MD = 2.601, SE = 0.668, *p* = 0.003 and MD = 2.582, SE = 0.703, *p* = 0.005, respectively). Figure 10 displays the analysis results of the Pe component.

## 4. Discussion

The main aim of the present study was to investigate the neural activity in complexity perception in visual processing. We hypothesized that, in the early stage of the visual processing, variations in artistic images’ complexity may not generate significant differences in P1, yet should induce significant differences in N1 amplitudes (H1). However, the results did not support the hypothesis. Regarding the participants’ behavior, we hypothesized that when they responded to the stimuli, the presence of response inhibition in the processing of visual complexity may be reflected in the form of N2 (H2). For the difference waves, we assumed that, in the objective observation, differences in complexity may cause processing in visual mismatch negativity (H3), and among the feedback-related ERPs, an automatic error identification-related ERP (error negativity) or a subsequent controlled error identification and task reassessment process (error positivity) may be found (H4).

The findings showed that complexity had little influence on VEPs related to early visual processing. The significant P1 and N1 in the right occipital area indicate asymmetrical variations in cortical neural activity during the early stages of processing complexity. The visual targets elicited a larger N2 over the anterior scalp, followed by a larger P3b, which called attention to the guidance attention to task-relevant stimuli. In this study, we found a significant vMMN, which may be explained by the potential relation to visual complexity perception processes. Finally, the Ne and Pe were elicited, revealing that the unaware errors were precipitated by lapses of attention relevant to visual complexity perception. The following provides further explanations.

### 4.1. P1 and N1

We discovered a significant P1 component in the occipital lobe, and the P1 amplitude in the right hemisphere was significantly higher than that in the left hemisphere. There was no significant difference between groups of different complexities, indicating that differences in complexity do not cause differences in P1 patterns. Although we discovered an N1 component, the mean magnitude of N1 in each of the three conditions was still a positive value, showing a weak activation. Therefore, we rejected the complexity-based visual selective attention allocation mentioned in hypothesis H1.

Early treatment of complexity may not create substantial variations in P1 amplitudes, but it should generate significant differences in N1 amplitudes, according to hypothesis H1. However, we discovered significant P1 following by a weak N1, which demonstrates that complexity gives rise to predictions of early attention, and that complexity differences do not give rise to changes in component magnitude; that is, in the early visual process, the variations in complexity have little effect on the allocation of attentional resources. The magnitude of components from this set, often referred to as “exogenous components”, can be modulated by attended spatial position or increasing the demand on visual discrimination of the stimulus. These spatial and nonspatial modulations of exogenous components are consistent with their interpretation in terms of a sensory enhancement mechanism that is relatively nonspecific with regard to individual features of stimuli, such as color and orientation [76].

The appearance of P1 has been linked to the capacity to detect stimuli in studies [77]. The P1 component was not triggered when the stimulus was warped beyond recognition. There have also been studies that indicated no change in the size of P1 when comparing known and unfamiliar things [78]. Differences in global complexity perception by P1 amplitude were eliminated in our investigation, confirming the idea that P1 represents early categorization based on global stimulus properties. The P1 amplitudes have similar magnitudes if the global stimulus properties are relatively similar across stimulus classes [20].

In some studies, complex stimuli cause changes in the amplitude of the N1, but in the attended cases, are modulated by space. In contrast, stimulus configuration modulated the amplitude of the N1 component, which was larger for complex stimuli than simple stimuli in both target and non-target conditions [41]. Among other image properties studied, high spatial frequency as an individual feature can cause a significant increasing in amplitudes with regard to the posterior N1 component [79]. In our study, complexity as an individual feature did not cause sensory enhancement mechanism in early visual processes, as reflected by the overall weakness of N1 amplitudes, and the fact that complexity differences did not cause significant differences in N1 amplitudes.

The magnitude of both P1 and N1 wave were unaffected by complexity, although the distinctions we discovered across hemispheres are worth discussing. Previous studies on emotion have explored hemispherical features, ambiguity differences between the left and right hemispheres, and degrees of reactivity to emotional and neutral stimuli [80,81]. The findings of this study may indicate asymmetrical variations in cortical neural activity during the early stages of processing complexity, with the right occipital lobe presumably taking the initiative.

### 4.2. N2

Previous studies have proven that visual stimulus of higher complexity can cause a larger anterior N2 amplitude by conducting tasks on numbers or shapes [16,82]. The anterior N2 usually refers to a negative-going wave with a frontal or central scalp maximum, corresponding to the findings of Pritchard et al. [45]. We can conclude that the anterior N2 is elicited by a visual stimulus with a high perceptual demand, which includes visual complexity as a general characteristic [43]. According to the previous studies, it will not be enough to cause a significant anterior N2 amplitude if the stimulus deviation is faint [83]. We discovered that somehow a greater complexity variation can induce significant anterior N2 amplitudes, which may explain why the N2 amplitudes between the high and medium groups, and the medium and low groups, are inconsequential. In addition, the anterior N2 also reflected a collection of processes broadly termed “cognitive control”, which was divined by an inhibition of a planned response [84]. In the experiment, the participants were required to discriminate the complexity level of the stimulus, and to make the option within a time limit of between 1800 and 2200 ms. The short reaction time may cause the tension in the participants and be another reason for the larger N2 amplitude in the frontal zone.

### 4.3. P3

The P3 wave was detected in the posterior parietal and occipital zones. We regard it as the P3b component caused by the attentional processing and target stimulus promotions in the oddball paradigm. According to the empirical and theoretical theories of P300, the P300 component may stem from neural inhibitory activity organized to delimit task-extraneous events to sculpt attentional focus and promote memory operations for target stimuli [85]. The task-relevant P3b potential is elicited during target stimulus processing. P3b reflects the match between the incoming stimulus and the voluntarily maintained attentional trace of the task-relevant stimulus [86,87]. The P3b component of the parietal zone we observed was the associated following-up component of the anterior N2, which represented the existence of attention-related processing on complexity and the participation in the task-relevant discrimination processes [88]. The P3b amplitude in the occipital zone was significantly higher than the parietal P3b wave under the three complexity conditions. However, no significant difference was detected between different complexity conditions. In a previous PET study, researchers reported parieto-occipital positivity (P300) for the intensity task. Therefore, we speculated that the larger occipital P3 wave voltage explained the pre-attention stimulus information to guide attention to task-related stimulus [89]. Attention processing related to complexity may originate more from the occipital zone, and P3b proved that processing complexity requires more attention.

### 4.4. Visual MMN

In this study, the results showed that the vMMN was related to visual complexity perception processes. Therefore, we speculate that the vMMN in this study followed a manifestation of active memory representations mentioned by Stefanics et al. [50]. The vMMN is often elicited by rare events embedded in a series of frequently repeating events. In the continuous display of low-complexity stimulus, the brain actively generates predictions of its sensory inputs using a generative model. As a characterization, the vMMN represents the deviation between the calculated predictions and the actual sensory inputs. This prediction-error account is currently thought to be the most reasonable account for generating the vMMN. For stimuli of different levels of complexity, participants had errors in their prediction and behaviors. In the experiment, the participants were asked to respond to high-complexity stimuli, which revealed that the vMMN was sensitive to intentional prediction [90,91].

In addition, compared with low-complexity stimulus, high-complexity stimulus aroused a larger amplitude of the vMMN. The result showed a positive correlation between the deviation in stimulus and the vMMN amplitude. This representation may be attributed to the further processing of complex stimulus abnormalities. Consistent with common findings in the auditory field, the perceptual discrimination performance is strongly associated with MMN characteristics, e.g., increasing stimulus deviance increases the MMN amplitude, which correlates with a higher discrimination rate [50]. It is worth noting that the difference between the deviated stimulus (high complexity) and the standard stimulus (low complexity) was ambiguous, whereas the vMMN amplitude remained at a significant level. However, the volatility of the vMMN in the frontal area reduced as the complexity difference grew, indicating that the high/low difference wave was the highest, the high/medium difference wave was the second-highest, and the medium/low difference wave was the lowest. We only discovered significant differences between the high/low and medium/low difference waves, which may be explained by the subtle difference between the adjacent groups, which would not be sufficient to attain statistical significance. Meanwhile, our research supported that the vMMN demonstrated automatic categorization processes based on fairly complex stimulus representation [63].

It has been reported that the neural generators of vMMN mainly include the occipito-temporal visual extrastriate areas (right hemisphere only) and the medial and lateral prefrontal areas (right-lateralized) [92]. Unlike previous studies, the vMMN found in our study had significantly different performances in the four zones, but had no significant characteristics of the right hemisphere. In this study, the vMMN caused by differences in visual complexity showed significant prefrontal volatility. Studies of the auditory MMN have implicated a role for the frontal lobe; the apparent variability in the location of the frontal source may stem from the variations in the degree of attentional focus on the stimuli [93]. Recent work that examined the oscillatory characteristics of the auditory MMN has demonstrated that the strength of frontal source responses is modulated by the active or passive nature of a task, in addition to stimulus complexity [94]. The vMMN, as a homolog of the auditory MMN, also has the potential role of frontal mechanisms [93,95]. Our findings can be explained by the pre-attentive change detection, given that the latest studies examined early inferior frontal cortex (IFC) mismatch response representing the effort in comparing a stimulus to the prediction [96]. The prefrontal neural generator was parallel to extensive visual memory and prediction research, which suggested that the prefrontal region plays a crucial role in encoding the temporal relationship between successive visual stimuli. According to the hierarchical predictive coding framework proposed by Friston [97,98,99], bottom-up forward connections convey prediction errors (MMN or mismatch response), and top-down backward connections carry predictions, which explain prediction errors (repetition suppression). In this study, the strong activities in the prefrontal area indicated the contribution to the prediction error response of visual complexity. The weaker vMMN amplitude of the occipital area may be explained by the fact that, instead of repeating the same stimulus, continuously exhibiting of nonredundant standard stimuli was the interpretation for the weak-repetition suppression effect [100].

### 4.5. Ne and Pe

To reveal the relationship between the ERP performance and the participants’ subjective behaviors of complexity perception, we also analyzed the difference wave of the error negative (Ne) and error positive (Pe) components.

The detection of errors is known to be associated with two successive neurophysiological components in EEG, with an early time-course following motor execution: the error-related negativity (ERN/Ne) [101,102,103] and late positivity (Pe) [65]. Within 100 ms of the error, Ne reflects the dynamic self-monitoring process in the medial frontal cortex. The reaction monitoring process can be located in the Anterior Cingulate Cortex (ACC) [102,104]. Compared with Ne, the Pe component appears after Ne and shows a more posterior and more central scalp distribution [65]. Our research found that the Ne wave had a significant amplitude at the Fz electrode, whereas the Pe wave appeared significantly on the FCz electrode. The characteristics of the scalp distribution in this study was in line with the previous research results.

Our experiment did not give feedback on the participants’ reactions. Therefore, the Ne wave and Pe wave found in the task were the neural manifestations of unconscious errors. As expected, incorrect reactions cause negative components, reflecting the error detection of the participants’ mismatch of the complexity stimulus with their experiences and expectations. The amplitude of the Ne component that we observed was small, which may be related to the limited reaction time and the participants generating pressure under the limit.

However, micro-negativity with similar onset also appeared after correct trials. Due to the oddball paradigm being selected instead of the Stroop task [105] or the Eriksen flanker task [106], the research did not comply with the characteristics of conflict monitoring [107]. Therefore, the tiny negative wave generated in the correct trials was interpreted as a small probability of guessing the correct response [65], which showed error detection in the negative ERP waveform, but showed correct responses in behavior.

A small number of related pieces of research on Pe have been conducted to date. Pe appears to index subsequent response monitoring processes such as error awareness [108]. Similarly, it has been suggested that Pe is related to error salience [65]. The amplitude of the Pe component that we observed was relatively large. Related to this, previous studies have shown that the amplitude of Pe is significantly related to the measure of the individual’s ability to successfully adapt to the speed demand during the experiment [109]. We speculate that after the participants adapted to the behavior of reacting to stimulus, the response time of correct behavior would be shortened, leading to the larger amplitude of the Pe wave.

In general, as an independent feature, complexity was not involved in modulation of the early visual components P1 and N1 on their amplitudes in the high-complexity condition. Instead, there was a significant right hemisphere response in all three conditions. Therefore, we speculated that complexity appears to be modulated by hemispheric asymmetry when processing artistic images at the early stage. Visual stimuli with higher complexity elicited larger anterior N2 amplitudes, but only produced significant differences between the high- and low-complexity groups, suggesting that larger differences in complexity were sufficient to modulate anterior N2 amplitude. The occipital features of the following-up P3b suggest that complexity-related attentional processing is more likely to originate in the occipital region. Furthermore, similar to the early visual components, complexity differences did not cause amplitude significance in P3b. In the high-complexity condition, the vMMN we discovered had frontal activation characteristics and larger amplitudes, indicating complexity differences were a factor to modulate vMMN amplitudes. Finally, Ne and Pe reflected error detection of complexity differences even under unconscious tasks.

## 5. Conclusions

Our work measured human neural responses to images of different visual complexity levels using the oddball paradigm, and preliminarily explored the neurocognitive responses of complexity perception in visual processing. In this study, we found that high-complexity stimuli did not stimulate significant neural activity in early visual processing, but it did evoke significant neural activity in the discrimination process. Features of the vMMN revealed that the prefrontal area indicated the contribution to the prediction error response of visual complexity, and the error negativity allowed for the unconscious error detection of mismatch in visual complexity stimulus and expectations.

This study is a preliminary exploration of the neural response to complexity involving several ERPs, which may overlap with each other. In follow-up research, we will develop a suitable experimental design and analysis method for each component. We took the stimuli for this visual complexity research from the SAVOIAS database to evaluate human brain activity for artistic images of various complexity. As a limitation of this study, it is still essential to expand the categories and quantities of stimuli in future studies to objectively describe the neural responses of visual complexity in general. This work has verified the significant vMMN features in processing visual complexity; our follow-up studies will use the combined paradigm of an equiprobable sequence and a traditional oddball sequence, and will control the occurrence and repetition probability of stimuli to more precisely describe the vMMN. In addition to the frequency of stimuli, we consider the physical energy delivered to the sensory system as a measure of stimuli, which had varying complexity ratings but contained the same physical energy [110]. Additionally, we will conduct experiments to source localization analysis, and subsequently use fMRI to describe the spatial information, orientation, and intensity information of neural activity sources that characterize the neural mechanisms involved in the identification of visual complexity.

In conclusion, our study did not investigate the contribution of the properties that may constitute the complexity of an image to the findings. Future research may be connected to recent discoveries in computer vision to refine the neural responses of specific image properties that make up computable image complexity to construct cognitively consistent neural network models [111,112] for use in areas such as image classification.

## Data Availability

The data presented in this study are available on request from the corresponding author.

## References

[1] Roach N.W., Hogben J.H. (2004). Attentional Modulation of Visual Processing in Adult Dyslexia: A Spatial-Cuing Deficit. Psychol. Sci..

[2] Standish R.K., Yang A., Shan Y. (2008). Concept and definition of complexity. Intelligent Complex Adaptive Systems.

[3] Koffka K. (2013). Principles of Gestalt Psychology.

[4] Berlyne D.E. (1958). The influence of complexity and novelty in visual figures on orienting responses. J. Exp. Psychol..

[5] Berlyne D.E. (1970). Novelty, complexity, and hedonic value. Percept. Psychophys..

[6] Oliva A., Mack M.L., Shrestha M., Peeper A. Identifying the perceptual dimensions of visual complexity of scenes. Proceedings of the 26th Annual Cognitive Science Society.

[7] Harper S., Michailidou E., Stevens R. (2009). Toward a definition of visual complexity as an implicit measure of cognitive load. ACM Trans. Appl. Percept..

[8] Nadal M., Munar E., Marty G., Cela-Conde C. (2010). Visual Complexity and Beauty Appreciation: Explaining the Divergence of Results. Empir. Stud. Arts.

[9] Da Silva M.P., Courboulay V., Estraillier P. Image complexity measure based on visual attention. Proceedings of the 18th IEEE International Conference on Image Processing.

[10] Wang Q.Z., Yang S., Liu M.L., Cao Z.K., Ma Q.G. (2014). An eye-tracking study of website complexity from cognitive load perspective. Decis. Support Syst..

[11] Coleman L.J., Elliott M.A. (2021). Disentangling cognitive from perceptual load using relational complexity. Vis. Cogn..

[12] Kriegeskorte N., Goebel R., Bandettini P. (2006). Information-based functional brain mapping. Proc. Natl. Acad. Sci. USA.

[13] Pfurtscheller G., Da Silva F.H.L. (1999). Event-related EEG/MEG synchronization and desynchronization: Basic principles. Clin. Neurophysiol..

[14] Thorpe S., Fize D., Marlot C. (1996). Speed of processing in the human visual system. Am. J. Ophthalmol..

[15] Cepeda-Freyre H.A., Garcia A.G., Eguibar J.R., Cortes C. (2020). Brain Processing of Complex Geometric Forms in a Visual Memory Task Increases P2 Amplitude. Brain Sci..

[16] Shigeto H., Ishiguro J., Nittono H. (2011). Effects of visual stimulus complexity on event-related brain potentials and viewing duration in a free-viewing task. Neurosci. Lett..

[17] Kursawe M.A., Zimmer H.D. (2015). Costs of storing colour and complex shape in visual working memory: Insights from pupil size and slow waves. Acta Psychol..

[18] Groen I.I.A., Jahfari S., Seijdel N., Ghebreab S., Lamme V.A.F., Scholte H.S. (2018). Scene complexity modulates degree of feedback activity during object detection in natural scenes. PLoS Comput. Biol..

[19] Luria R., Sessa P., Gotler A., Jolicoeur P., Dell’Acqua R. (2010). Visual Short-term Memory Capacity for Simple and Complex Objects. J. Cogn. Neurosci..

[20] Klimesch W. (2011). Evoked alpha and early access to the knowledge system: The P1 inhibition timing hypothesis. Brain Res..

[21] Rugg M.D., Coles M.G.H. (1995). Electrophysiology of mind. Event-Related Brain Potentials and Cognition.

[22] Luck S.J. (2005). An Introduction to the Event-related Technique.

[23] Di Russo F., Martinez A., Sereno M.I., Pitzalis S., Hillyard S.A. (2002). Cortical sources of the early components of the visual evoked potential. Hum. Brain Mapp..

[24] Foxe J.J., Simpson G.V. (2002). Flow of activation from V1 to frontal cortex in humans-A framework for defining "early" visual processing. Exp. Brain Res..

[25] Schindler S., Bublatzky F. (2020). Attention and emotion: An integrative review of emotional face processing as a function of attention. Cortex.

[26] De Haan M., Johnson M.H., Halit H. (2003). Development of face-sensitive event-related potentials during infancy: A review. Int. J. Psychophysiol..

[27] Goffaux V., Gauthier I., Rossion B. (2003). Spatial scale contribution to early visual differences between face and object processing. Cogn. Brain Res..

[28] Taylor M.J., Batty M., Itier R.J. (2004). The faces of development: A review of face processing in early childhood. J. Cogn. Neurosci..

[29] Itier R.J., Taylor M.J. (2004). N170 or N1? Spatiotemporal differences between object and face processing using ERPs. Cereb. Cortex.

[30] Rossion B., Caharel S. (2011). ERP evidence for the speed of face categorization in the human brain: Disentangling the contribution of low-level visual cues from face perception. Vis. Res..

[31] Herrmann C.S., Knight R.T. (2001). Mechanisms of human attention: Event-related potentials and oscillations. Neurosci. Biobehav. Rev..

[32] Luck S.J., Heinze H.J., Mangun G.R., Hillyard S.A. (1990). Visual event-related potentials index focused attention within bilateral stimulus arrays. ii. Functional dissociation of P1 and N1 components. Electroencephalogr. Clin. Neurophysiol..

[33] Luck S.J., Ford M.A. (1998). On the role of selective attention in visual perception. Proc. Natl. Acad. Sci. USA.

[34] Ángel C., Juan L., Eduardo M., Pío T. (2006). Temporal attention enhances early visual processing: A review and new evidence from event-related potentials. Brain Res..

[35] Zhang W., Luck S.J. (2009). Feature-based attention modulates feedforward visual processing. Nat. Neurosci..

[36] Righi S., Orlando V., Marzi T. (2014). Attractiveness and affordance shape tools neural coding: Insight from ERPs. Int. J. Psychophysiol..

[37] He X., Fan S.L., Zhou K., Chen L. (2004). Cue Validity and Object-Based Attention. J. Cogn. Neurosci..

[38] He X., Humphreys G., Fan S.L., Chen L., Han S.H. (2008). Differentiating spatial and object-based effects on attention: An event-related brain potential study with peripheral cueing. Brain Res..

[39] Vogel E.K., Luck S.J. (2000). The visual N1 component as an index of a discrimination process. Psychophysiology.

[40] Hopf J.M., Vogel E., Woodman G., Heinze H.J., Luck S.J. (2002). Localizing Visual Discrimination Processes in Time and Space. J. Neurophysiol..

[41] Perri R.L., Berchicci M., Bianco V., Quinzi F., Spinelli D., Di Russo F. (2019). Perceptual load in decision making: The role of anterior insula and visual areas. An ERP study. Neuropsychologia.

[42] Doherty J.R. (2005). Synergistic effect of combined temporal and spatial expectations on visual attention. J. Neurosci. Off. J. Soc. Neurosci..

[43] Folstein J.R., Petten C.V. (2008). Influence of cognitive control and mismatch on the N2 component of the ERP: A review. Psychophysiology.

[44] Näätänen R., Gaillard A.W.K. (1983). The orienting reflex and the N2 deflection of the event related potential (ERP). Adv. Psychol..

[45] Pritchard W.S., Shappell S.A., Brandt M.E. (1991). Psychophysiology of N200/N400: A review and classification scheme. Adv. Psychophysiol..

[46] Ritter W., Simson R., Vaughan H.G., Friedman D. (1979). A brain event related to the making of a sensory discrimination. Science.

[47] Borchard J.P., Barry R.J., Blasio F.D. (2015). Sequential processing in an auditory equiprobable Go/NoGo task with variable interstimulus interval. Int. J. Psychophysiol..

[48] Ritter W., Simson R., Vaughan H.G., Macht M. (1982). Manipulation of event-related potential manifestations of information processing stages. Science.

[49] Courchesne E., Hillyard S.A., Galambos R. (1975). Stimulus novelty, task relevance and the visual evoked potential in man. Electroencephalogr. Clin. Neurophysiol..

[50] Stefanics G., Kremlacek J., Czigler I. (2014). Visual mismatch negativity: A predictive coding view. Front. Hum. Neurosci..

[51] Garrido M.I., Friston K.J., Kiebel S.J., Stephan K.E., Baldeweg T., Kilner J.M. (2008). The functional anatomy of the MMN: A DCM study of the roving paradigm. Neuroimage.

[52] Stefanics G., Czigler I. (2012). Automatic prediction error response to hands with unexpected laterality: An electrophysiological study. Neuroimage.

[53] Astikainen P., Lillstrang E., Ruusuvirta T. (2008). Visual mismatch negativity for changes in orientation—A sensory memory-dependent response. Eur. J. Neurosci..

[54] Kimura M., Katayama J., Ohira H., Schroger E. (2009). Visual mismatch negativity: New evidence from the equiprobable paradigm. Psychophysiology.

[55] Kimura M., Schroger E., Czigler I., Ohira H. (2010). Human visual system automatically encodes sequential regularities of discrete events. J. Cogn. Neurosci..

[56] Czigler I., Balázs L., Winkler I. (2002). Memory-based detection of task-irrelevant visual changes. Psychophysiology.

[57] Czigler I., Weisz J., Winkler I. (2007). Backward masking and visual mismatch negativity: Electrophysiological evidence for memory-based detection of deviant stimuli. Psychophysiology.

[58] Stefanics G., Kimura M., Czigler I. (2011). Visual mismatch negativity reveals automatic detection of sequential regularity violation. Front. Hum. Neurosci..

[59] File D., File B., Bodnar F., Sulykos I., Kecskes-Kovacs K., Czigler I. (2007). Visual mismatch negativity (vMMN) for low- and high-level deviances: A control study. Atten. Percept. Psychophys..

[60] Kojouharova P., File D., Sulykos I., Czigler I. (2019). Visual mismatch negativity and stimulus-specific adaptation: The role of stimulus complexity. Exp. Brain Res..

[61] Kujala T., Tervaniemi M., Schröger E. (2007). The mismatch negativity in cognitive and clinical neuroscience: Theoretical and methodological considerations. Biol. Psychol..

[62] Kujala T., Näätänen R. (2010). The adaptive brain: A neurophysiological perspective. Prog. Neurobiol..

[63] Czigler I. (2013). Visual mismatch negativity and categorization. Brain Topogr..

[64] Beck A.K., Berti S., Czernochowski D., Lachmann T. (2021). Do categorical representations modulate early automatic visual processing? A visual mismatch-negativity study. Biol. Psychol..

[65] Falkenstein M., Hoormann J., Christ S., Hohnsbein J. (2000). ERP components on reaction errors and their functional significance: A tutorial. Biol. Psychol..

[66] Falkenstein M., Hohnsbein J., Hoormann J., Blanke L. (1990). Effects of errors in choice reaction tasks on the ERP under focused and divided attention. Psychophysiological Brain Res..

[67] Kopp B., Rist F., Mattler U. (1996). N200 in the flanker task as a neurobehavioral tool for investigating executive control. Psychophysiology.

[68] Van Veen V., Carter C.S. (2002). The timing of action-monitoring processes in the anterior cingulate cortex. J. Cogn. Neurosci..

[69] Frank M.J., Woroch B.S., Curran T. (2005). Error-related negativity predictions reinforcement learning and conflict biases. Neuron.

[70] Elham S., Mona J., Margrit B. (2020). Visual complexity analysis using deep intermediate-layer features. Comput. Vis. Image Underst..

[71] Deiber M.P., Hasler R., Colin J., Dayer A., Aubry J.M., Baggio S., Perroud N., Ros T. (2020). Linking alpha oscillations, attention and inhibitory control in adult ADHD with EEG neurofeedback. NeuroImage Clin..

[72] Delorme A., Makeig S. (2004). EEGLAB: An open source toolbox for analysis of single-trial EEG dynamics including independent component analysis. J. Neurosci. Methods.

[73] Lopez-Calderon J., Luck S.J. (2014). ERPLAB: An open-source toolbox for the analysis of event-related potentials. Front. Hum. Neurosci..

[74] Kimura M. (2012). Visual mismatch negativity and unintentional temporal-context-based prediction in vision. Int. J. Psychophysiol..

[75] Bernstein P.S., Scheffers M.K. (1995). “Where did I go wrong?” A psychophysiological analysis of error detection. J. Exp. Psychol. Hum. Percept. Perform..

[76] Wills A.J., Lavric A., Croft G.S., Hodgson T.L. (2007). Predictive learning, prediction errors, and attention: Evidence from event-related potentials and eye tracking. J. Cogn. Neurosci..

[77] Freunberger R., Klimesch W., Griesmayr B., Sauseng P., Gruber W. (2008). Alpha phase coupling reflects object recognition. Neuroimage.

[78] Busch N.A., Herrmann C.S., Müller M.M., Lenz D., Gruber T. (2006). A cross-laboratory study of event-related gamma activity in a standard object recognition paradigm. Neuroimage.

[79] Rokszin A.A., Gyori-Dani D., Nyul L.G., Csifcsak G. (2016). Electrophysiological correlates of top-down effects facilitating natural image categorization are disrupted by the attenuation of low spatial frequency information. International. J. Psychophysiol..

[80] Mattavelli G., Rosanova M., Casali A.G., Papagno C., Lauro L.J.R. (2016). Timing of emotion representation in right and left occipital region: Evidence from combined TMS-EEG. Brain Cogn..

[81] Prete G., Capotosto P., Zappasodi F., Tommasi L. (2018). Contrasting Hemispheric Asymmetries for Emotional Processing From Event-Related Potentials and Behavioral Responses. Neuropsychology.

[82] Daffner K.R., Mesulam M.M., Scinto L.F.M., Calvo V., Faust R., Holcomb P.J. (2000). An electrophysiological index of stimulus unfamiliarity. Psychophysiology.

[83] Maekawa T., Goto Y., Kinukawa N., Taniwaki T., Kanba S., Tobimatsu S. (2005). Functional characterization of mismatch negativity to a visual stimulus. Clin. Neurophysiol..

[84] Bruin K.J., Wijers A.A. (2002). Inhibition, response mode, and stimulus probability: A comparative event-related potential study. Clin. Neurophysiol..

[85] Polich J. (2007). Updating P300: An integrative theory of P3a and P3b. Clin. Neurophysiol..

[86] Katayama J., Polich J. (1998). Stimulus context determines P3a and P3b. Psychophysiology.

[87] Goldstein A., Spencer K.M., Donchin E. (2002). The influence of stimulus deviance and novelty on the P300 and novelty P3. Psychophysiology.

[88] Volpe U., Mucci A., Bucci P., Merlotti E., Galderisi S., Maj M. (2007). The cortical generators of P3a and P3b: A LORETA study. Brain Res. Bull..

[89] Luck S.J., Hillyard S.A. (1994). Electrophysiological correlates of feature analysis during visual search. Psychophysiology.

[90] Kimura M., Ohira H., Schroger E. (2010). Localizing sensory and cognitive systems for pre-attentive visual deviance detection: An sLORETA analysis of the data of Kimura et al. (2009). Neurosci. Lett..

[91] Bubic A., Bendixen A., Schubotz R.I., Jacobsen T., Schroger E. (2010). Differences in processing violations of sequential and feature regularities as revealed by visual event-related brain potentials. Brain Res..

[92] Kimura M., Kondo H., Ohira H., Schroger E. (2012). Unintentional temporal-context-based prediction of emotional faces: An electrophysiological study. Cereb. Cortex.

[93] Hedge C., Stothart G., Jones J.T., Frias P.R., Magee K.L., Brooks J.C.W. (2015). A frontal attention mechanism in the visual mismatch negativity. Behav. Brain Res..

[94] MacLean S.E., Ward L.M. (2014). Temporo-frontal phase synchronization supports hierarchical network for mismatch negativity. Clin. Neurophysiol..

[95] Chen Y., Huang X.T., Luo Y.M., Peng C.H., Liu C.X. (2010). Differences in the neural basis of automatic auditory and visual time perception: ERP evidence from an across-modal delayed response oddball task. Brain Res..

[96] Tse C.Y., Shum Y.H., Wang Y. (2021). Fronto-occipital mismatch responses in pre-attentive detection of visual changes: Implication on a generic brain network underlying Mismatch Negativity (MMN). Neuroimage.

[97] Karl F., James K., Harrison L. (2006). A free energy principle for the brain. J. Physiol. -Paris.

[98] Friston K. (2008). Hierarchical models in the brain. PLoS Comput. Biol..

[99] Friston K. (2012). The free-energy principle: A unified brain theory?. Nat. Rev. Neurosci..

[100] Claudia M., Gyula K., Catarina A., Gregor U., Hayn L., Christoph R. (2018). Visual mismatch negativity indicates automatic, task-independent detection of artistic image composition in abstract artworks. Biol. Psychol..

[101] Gehring W., Coles M., Meyer D., Donchin E. (1990). The error-related negativity: An event-related brain potential accompanying errors. Psychophysiology.

[102] William J., Gehring B.G., Michael G.H., Coles D.E., Meyer E.D. (1993). A Neural System for Error Detection and Compensation. Psychol. Sci..

[103] Falkenstein M., Hohnsbein J., Hoormann J., Blanke L. (1991). Effects of crossmodal divided attention on late ERP components. II. Error processing in choice reaction tasks. Electroencephalogr. Clin. Neurophysiol..

[104] Vincent V.V., Cameron S.C. (2002). The anterior cingulate as a conflict monitor: fMRI and ERP studies. Physiol. Behav..

[105] MacLeod C.M. (1991). Half a century of research on the Stroop effect: An integrative review. Psychol. Bull..

[106] Gratton G., Coles M.G., Donchin E. (1992). Optimizing the use of information: Strategic control of activation of responses. J. Exp. Psychol..

[107] Kerns J.G., Cohen J.D., MacDonald A.W., Cho R.Y., Stenger V. (2004). Andrew and Carter Cameron, S. Anterior Cingulate Conflict Monitoring and Adjustments in Control. Science.

[108] Sander N.K., Richard R., Jos B., Guido P.H.B., Albert K. (2001). Error-related brain potentials are differentially related to awareness of response errors: Evidence from an antisaccade task. Psychophysiology.

[109] Roland V., Gilles P., Patrik V. (2008). Unavoidable errors: A spatio-temporal analysis of time-course and neural sources of evoked potentials associated with error processing in a speeded task. Neuropsychologia.

[110] Beck A.K., Czernochowski D., Lachmann T., Berti S. (2021). Do categorical representations modulate early perceptual or later cognitive visual processing? An ERP study. Brain Cogn..

[111] Qin Y., Zhan Y., Wang C.M., Zhang J.C., Yao L., Guo X.J., Wu X., Hu B. (2016). Classifying four-category visual objects using multiple ERP components in single-trial ERP. Cogn. Neurodynamics.

[112] Lawhern V.J., Solon A.J., Waytowich N.R., Gordon S.M., Hung C.P., Lance B.J. (2018). EEGNet: A compact convolutional neural network for EEG-based brain–computer interfaces. J. Neural Eng..

